# Pro-thrombotic changes associated with exposure to ambient ultrafine particles in patients with chronic obstructive pulmonary disease: roles of lipid peroxidation and systemic inflammation

**DOI:** 10.1186/s12989-022-00503-9

**Published:** 2022-10-24

**Authors:** Teng Wang, Xi Chen, Haonan Li, Wu Chen, Yifan Xu, Yuan Yao, Hanxiyue Zhang, Yiqun Han, Lina Zhang, Chengli Que, Jicheng Gong, Xinghua Qiu, Tong Zhu

**Affiliations:** 1 BIC-ESAT and SKL-ESPC, College of Environmental Sciences and Engineering, Peking University, Beijing, China; 2 Hebei Technology Innovation Center of Human Settlement in Green Building (TCHS), Shenzhen Institute of Building Research Co., Ltd., Xiongan, China; 3 Environmental Research Group, MRC Centre for Environment and Health, Imperial College London, London, UK; 4 Shi Cha Hai Community Health Service Center, Beijing, China; 5 Peking University First Hospital, Peking University, Beijing, China

**Keywords:** Particulate matter, Ultrafine particles, COPD, Platelet activation, Thrombosis, Lipid peroxidation, Inflammation

## Abstract

**Background:**

Exposure to particulate matter air pollution is associated with an increased risk of cardiovascular mortality in patients with chronic obstructive pulmonary disease (COPD), but the underlying mechanisms are not yet understood. Enhanced platelet and pro-thrombotic activity in COPD patients may explain their increased cardiovascular risk. We aim to explore whether short-term exposure to ambient particulate matter is associated with pro-thrombotic changes in adults with and without COPD, and investigate the underlying biological mechanisms in a longitudinal panel study. Serum concentration of thromboxane (Tx)B2 was measured to reflect platelet and pro-thrombotic activity. Lipoxygenase-mediated lipid peroxidation products (hydroxyeicosatetraenoic acids [HETEs]) and inflammatory biomarkers (interleukins [ILs], monocyte chemoattractant protein-1 [MCP-1], tumour necrosis factor alpha [TNF-α], and macrophage inflammatory proteins [MIPs]) were measured as potential mediating determinants of particle-associated pro-thrombotic changes.

**Results:**

53 COPD and 82 non-COPD individuals were followed-up on a maximum of four visits conducted from August 2016 to September 2017 in Beijing, China. Compared to non-COPD individuals, the association between exposure to ambient ultrafine particles (UFPs) during the 3–8 days preceding clinical visits and the TxB2 serum concentration was significantly stronger in COPD patients. For example, a 10^3^/cm^3^ increase in the 6-day average UFP level was associated with a 25.4% increase in the TxB2 level in the COPD group but only an 11.2% increase in the non-COPD group. The association in the COPD group remained robust after adjustment for the levels of fine particulate matter and gaseous pollutants. Compared to the non-COPD group, the COPD group also showed greater increases in the serum concentrations of 12-HETE (16.6% vs. 6.5%) and 15-HETE (9.3% vs. 4.5%) per 10^3^/cm^3^ increase in the 6-day UFP average. The two lipid peroxidation products mediated 35% and 33% of the UFP-associated increase in the TxB2 level of COPD patients. UFP exposure was also associated with the increased levels of IL-8, MCP-1, MIP-1α, MIP-1β, TNF-α, and IL-1β in COPD patients, but these inflammatory biomarkers did not mediate the TxB2 increase.

**Conclusions:**

Short-term exposure to ambient UFPs was associated with a greater pro-thrombotic change among patients with COPD, at least partially driven by lipoxygenase-mediated pathways following exposure.

*Trial registration* ChiCTR1900023692. Date of registration June 7, 2019, i.e. retrospectively registered.

**Supplementary Information:**

The online version contains supplementary material available at 10.1186/s12989-022-00503-9.

## Background

Air pollution, especially particulate matter (PM), is an important risk factor related to both chronic obstructive pulmonary disease (COPD) and cardiovascular diseases (CVDs) [1–5]. It is well-established that COPD is linked to various cardiovascular comorbidities including cardiac arrhythmia, acute myocardial infarction (MI), congestive heart failure, stroke, and thromboembolism [6–10]; approximately 30–50% of COPD deaths are caused by CVDs [11]. Given the higher risk of CVDs in people with COPD, they may be more susceptible to air pollution exposure than healthier people [12]. A recent cohort study found that long-term PM exposure was associated with an increased risk of CVD mortality among adults with COPD [13]. However, the biological mechanisms behind the epidemiological observations are unclear.

Platelets are critical for hemostasis, thrombosis, and subsequent MI and stroke [14]. Platelet and pro-thrombotic activity increases early in COPD patients and rises further during acute exacerbations, explaining the enhanced cardiovascular mortality risk [11, 15, 16]. Short-term exposure to ambient PM, in particular ultrafine particles (UFPs) or diesel exhaust particles (DEPs), activates platelets and enhances thrombus formation in experimental animals [17, 18] and in subjects with cardiometabolic disorders including coronary heart disease [19, 20], diabetes [21], and obesity [22]. However, few studies on COPD patients have appeared. We and other groups have reported that short-term exposure to PM is associated with biomarkers of lipid peroxidation and systemic inflammation in COPD patients [12, 23–26], which might trigger platelet activation and thrombosis [27, 28]. Thus, we hypothesized that short-term exposure to PM would be associated with pro-thrombotic changes in patients with COPD.

Thromboxane (Tx)A2, a pro-thrombotic prostanoid, activates platelets and induces irreversible platelet aggregation during primary haemostasis and atherothrombosis [14]. It is synthesised by cyclooxygenase (COX)-1 and released from activated platelets. Estimates of platelet TxA2 biosynthesis are obtained via ex vivo measurements of its chemically stable hydrolysis product TxB2 that can be easily measured in serum as a sensitive and specific biomarker of platelet COX-1 activity [29]. Serum TxB2 has been used extensively to assess the human pharmacology of platelet COX-1 inhibition in health and disease [30]. In this study, we explored whether short-term exposure to ambient UFPs or fine particulate matter (PM_2.5_) may increase serum TxB2 concentration in COPD patients and non-COPD controls. We also measured the serum levels of biomarkers of lipoxygenase-mediated lipid peroxidation (hydroxyeicosatetraenoic acids [HETEs]) and inflammation (interleukins [ILs], monocyte chemoattractant protein-1 [MCP-1], tumour necrosis factor alpha [TNF-α], and macrophage inflammatory proteins [MIPs]) to explore if these might mediate the TxB2 change associated with PM exposure.

## Results

### Descriptive statistics

We included 53 COPD and 82 non-COPD individuals (Table 1). Among the 53 COPD patients, 17 (32%) were mild, 27 (51%) were moderate, and 9 (17%) were severe or very severe (Additional file 1: Table S1). Compared to the non-COPD group, COPD patients evidenced markedly poorer lung function as reflected by the FEV_1_/FVC ratio (*P* < 0.001). The proportion of participants who took aspirin was similar between COPD and non-COPD group (24.5% vs. 26.8%; *P* = 0.92). The serum TxB2 level was not significant between COPD and non-COPD group (*P* = 0.59), but it appeared higher in the severe COPD patients compared to the moderate (*P* = 0.1; Additional file 1: Figure S1). Regardless of COPD severity, taking aspirin significantly reduced TxB2 concentrations (Additional file 1: Figure S1). Ambient air pollutant concentrations are summarized in Additional file 1: Table S2. The daily mean (standard deviation) concentrations of PM_2.5_ and UFPs were 69.7 (61.1) μg/m^3^ and 12.5 × 10^3^ (4.3 × 10^3^) counts/cm^3^ respectively. The PM_2.5_ and UFP levels were not correlated (Spearman correlation coefficient *r* = − 0.03).

**Table 1:** Summary of the participant characteristics and biomarkers

	COPD	Non-COPD	*P*^c^
Characteristics			
No. of participants	53	82	
No. of clinical visits	153	256	
Age, mean (SD), y	64.7 (6.6)	58.2 (7.4)	< 0.001
Body mass index, mean (SD), kg/m^2^	24.5 (3.2)	24.8 (3.4)	0.57
Male, No. (%)	30 (56.6%)	26 (31.7%)	0.007
High school or above, No. (%)	31 (58.5%)	47 (57.3%)	0.84
Monthly salary ≥ 5000 CNY, No. (%)	8 (15.1%)	13 (15.9%)	0.32
FEV_1_/FVC ratio, mean (SD), %	56.5 (12.8)	76.4 (5.4)	< 0.001
COPD medication use^a^, No. (%)	91 (59.5%)	0 (0%)	< 0.001
Aspirin use^a^, No. (%)	13 (24.5%)	22 (26.8%)	0.92
Biomarkers^b^, geometric mean (IQR)			
TxB2, ng/mL	8.5 (4.2, 29.0)	7.1 (5.1, 27.9)	0.59
12-HETE, ng/mL	24.7 (14.7, 50.2)	27.3 (18.4, 51.0)	0.59
15-HETE, ng/mL	0.8 (0.5, 1.4)	0.8 (0.5, 1.3)	0.69
IL-1β, pg/mL	1.1 (0.6, 1.3)	1.0 (0.6, 1.2)	0.46
IL-8, pg/mL	13.6 (8.0, 22.7)	15.3 (8.5, 24.0)	0.45
MCP-1, ng/mL	0.48 (0.41, 0.64)	0.48 (0.41, 0.62)	0.96
MIP-1α, pg/mL	7.3 (1.4, 19.0)	7.1 (1.8, 19.6)	0.86
MIP-1β, pg/mL	36.0 (28.6, 56.4)	38.6 (26.8, 67.1)	0.66
TNF-α, pg/mL	10.5 (7.0, 13.8)	11.4 (7.4, 16.6)	0.43

FEV_1_, forced expiratory volume in 1 s; FVC, forced vital capacity; TxB2, thromboxane B2; HETE, hydroxyeicosatetraenoic acid; IL-1β, interleukin 1 beta; IL-8, interleukin-8; MCP-1, monocyte chemoattractant protein-1; MIP-1α, macrophage inflammatory protein 1 alpha; MIP-1β, macrophage inflammatory protein 1 beta; TNF-α, tumour necrosis factor alpha

^a^Information on medication use was recorded on baseline questionnaires and multiple-checked by follow-up questionnaires

^b^Shown are geometric means (interquartile ranges) of each participant's average level across all repeated measurements

^c^*P* values were calculated using *t*-test or chi-square test when appropriate

### Association between ambient air particulate matter and serum TxB2 levels

**Fig. 1:**
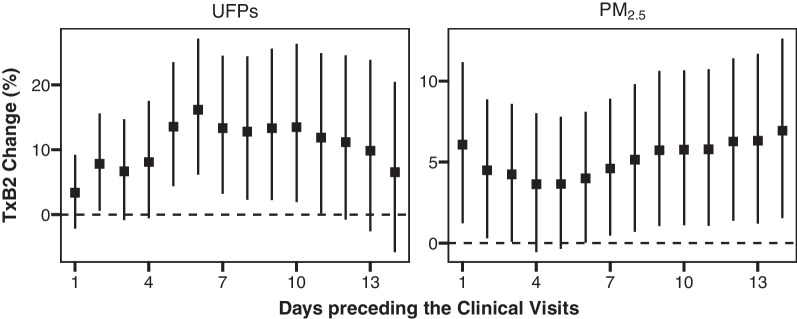
Association between ambient air particulate matter and serum thromboxane (Tx)B2 levels in all participants. Shown are percent changes (95% CIs) in serum TxB2 levels per 10^3^/cm^3^ increase in UFPs and per 10 μg/m^3^ increase in PM_2.5_ during the 1–14 days preceding the clinical visits. The estimates were obtained using linear mixed-effects models adjusted for age, sex, the body mass index, educational level, income, smoking status, temperature, relative humidity, and the day of the week

All participants exhibited significant increases in serum TxB2 levels as the average concentrations of UFPs and PM_2.5_ increased over the 1–14 days preceding the clinical visits (Fig. 1). The TxB2 changes associated with particle exposure peaked in a 6-day time window for UFPs (16.2% [95% CI 6.2–27.1%] increase per 10^3^/cm^3^) and a 14-day time window for PM_2.5_ (6.9% [95% CI 1.5–12.6%] increase per 10 µg/m^3^).

**Fig. 2:**
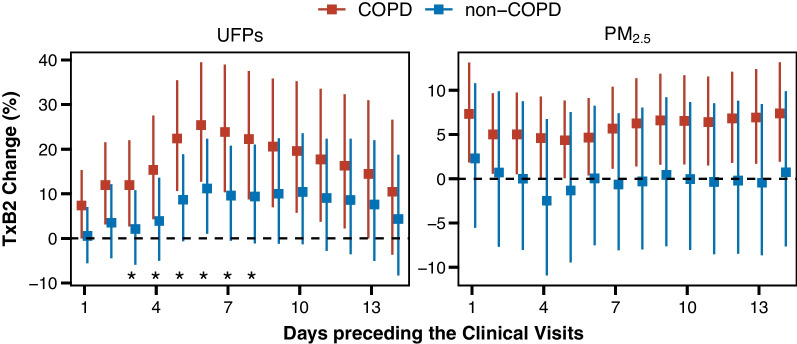
Association between ambient air particulate matter and serum thromboxane (Tx)B2 levels modified by COPD status. Shown are percent changes (95% CIs) in serum TxB2 levels per 10^3^/cm^3^ increase in UFPs and per 10 μg/m^3^ increase in PM_2.5_ during the 1–14 days preceding the clinical visits. Based on the linear mixed-effects models, interaction terms (COPD status and pollutant concentration) were added to calculate the estimates within each group and *P*_*interaction*_ for the effect modification. The significant between-group differences with *P*_*interaction*_ < 0.05 were indicated by asterisks

Figure 2 shows that the associations between the serum TxB2 level and the ambient UFP and PM_2.5_ levels were consistently stronger in the COPD than the non-COPD group. The UFP-associated between-group differences in the TxB2 change were significant in the 3- to 8-day analyses. For example, a 10^3^/cm^3^ increase in the 6-day average UFP level was associated with a 25.4% (95% CI 12.8–39.4%) increase in the TxB2 level in the COPD group but only an 11.2% (95% CI 1.1–22.3%) increase in the non-COPD group. The PM_2.5_-associated increase in TxB2 was significant only in the COPD group; the largest estimate was 7.4% (95% CI 1.9–13.1%) per 10 µg/m^3^ increase for the 14-day PM_2.5_ average.

Table 2 shows the robustness of the serum TxB2 changes with the UFP and PM_2.5_ levels in the two-pollutant models. In the COPD group, adjustment for PM_2.5_ or the gaseous pollutants changed the association between the UFP and TxB2 levels only slightly, but the significance of associations between PM_2.5_ and TxB2 was changed toward null after adjusting for UFPs or the gaseous pollutants. The UFP-associated TxB2 increase in the COPD group remained stable in sensitivity analyses (Additional file 1: Table S3), including in participants who had urinary cotinine levels < 50 ng/mL (n = 112, visit = 338; *P* = 0.009); those who were not TxB2 outliers (129, 384 visits; *P* < 0.001); those who visited in warm weather (135, 297 visits; *P* < 0.001); and those restricting to the first three visits (135, 342 visits; *P* < 0.001). On the contrary, the UFP-associated estimates in the non-COPD group were appreciably attenuated after adjustment for the SO_2_ and CO levels and in the sensitivity analyses (Tables 2 and S3). Overall, the association between the UFPs and serum TxB2 concentrations in the COPD group was robust.

**Table 2:** Single-pollutant and two-pollutant models for the association between ambient air particulate matter and serum thromboxane B2 levels

Model	COPD		Non-COPD		*P*_*interaction*_
	Change, % (95% CI)	*P*	Change, % (95% CI)	*P*	
UFPs	25.4 (12.8, 39.4)	< 0.001	11.2 (1.1, 22.3)	0.03	0.01
UFPs + PM_2.5_	22.4 (9.2, 37.3)	< 0.001	10.5 (0.4, 21.7)	0.04	0.04
UFPs + NO_2_	23.6 (9.9, 38.9)	< 0.001	11 (0.8, 22.3)	0.04	0.03
UFPs + SO_2_	22.3 (9.1, 37.1)	< 0.001	8.9 (− 1.7, 20.6)	0.10	0.01
UFPs + CO	23.1 (10.2, 37.6)	< 0.001	10 (− 0.2, 21.2)	0.06	0.02
UFPs + O_3_	25.7 (13, 39.8)	< 0.001	12.4 (1.9, 24)	0.02	0.02
PM_2.5_	7.4 (1.9, 13.1)	0.008	0.7 (− 7.6, 9.9)	0.87	0.11
PM_2.5_ + UFPs	5.7 (0.3, 11.4)	0.04	− 0.4 (− 8.6, 8.5)	0.93	0.13
PM_2.5_ + NO_2_	5.1 (− 4.6, 15.8)	0.31	− 1 (− 11.9, 11.2)	0.86	0.13
PM_2.5_ + SO_2_	3.9 (− 2.7, 11)	0.25	− 3.8 (− 13.3, 6.7)	0.46	0.06
PM_2.5_ + CO	4.1 (− 4, 12.8)	0.33	− 3.6 (− 14.7, 8.9)	0.56	0.07
PM_2.5_ + O_3_	7.5 (1.5, 13.7)	0.01	0.8 (− 7.8, 10.1)	0.86	0.11

Shown are percent changes (95% CIs) in serum TxB2 levels per 10^3^/cm^3^ increase in the 6-day UFP average and per 10 μg/m^3^ increase in the 14-day PM_2.5_ average

### Mediation roles of lipid peroxidation and inflammation

**Fig. 3:**
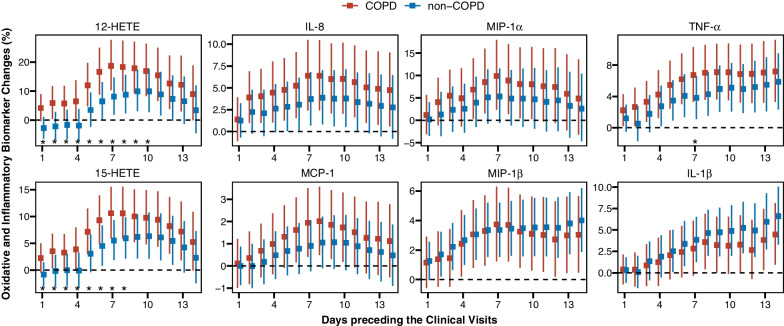
Associations between air ultrafine particle (UFP) levels and biomarkers of lipid peroxidation and inflammation modified by COPD status. Shown are percent changes (95% CIs) in serum biomarker levels per 10^3^/cm^3^ increase in UFP levels during the 1–14 days preceding the clinical visits. The significant between-group differences in UFP-associated biomarker changes with *P*_*interaction*_ < 0.05 were indicated by asterisks

To explore potential biological mechanisms underlying the UFP-associated TxB2 increase in the COPD group, we evaluated the associations between UFP levels and biomarkers of lipid peroxidation (HETEs) and inflammation (IL-1β, IL-8, MCP-1, MIP-1α, MIP-1β, and TNF-α) and conducted mediation analyses. COPD status significantly modified the associations between UFP and HETE levels (Fig. 3). For each 10^3^/cm^3^ increase in the 6-day UFP average, the 12-HETE and 15-HETE levels increased by 16.6% (95% CI 8.8–25%) and 9.3% (95% CI 4.9–13.9%) in the COPD group, but by only 6.5% (95% CI 0.3–13.1%) and 4.5% (95% CI 0.8–8.3%) in the non-COPD group. Among the inflammatory biomarkers, UFP exposure was associated with greater increases in the concentrations of IL-8, MCP-1, MIP-1α, and TNF-α in the COPD group, compared to the non-COPD group, but the between-group differences were not statistically significant except TNF-α.

As shown in Table 3, 12-HETE and 15-HETE mediated 35% (95% CI 6–72%) and 33% (95% CI 7–56%) of the UFP-associated increase in the TxB2 level in the COPD group but no mediation was apparent in the non-COPD group. None of the inflammatory mediators evidenced mediation effect on the association between UFP exposure and TxB2.

**Table 3:** The mediation effects of lipid peroxidation and inflammation on the ultrafine particles (UFP)-associated thromboxane B2 increase in the participants with and without COPD

Mediators	COPD		Non-COPD	
	Mediation proportions (95% CI)	*P*	Mediation proportions (95% CI)	*P*
12-HETE	0.35 (0.06, 0.72)	0.04	0.61 (− 3.48, 2.16)	0.46
15-HETE	0.33 (0.07, 0.56)	0.02	0.8 (− 4.86, 6.36)	0.42
IL-1β	0 (− 0.04, 0.07)	0.86	− 0.02 (− 0.44, 0.65)	0.70
IL-8	0 (− 0.08, 0.07)	0.86	0.12 (− 1.04, 2.81)	0.54
MCP-1	0.03 (− 0.07, 0.13)	0.28	0.01 (− 0.73, 0.37)	0.78
MIP-1α	0.03 (− 0.04, 0.16)	0.36	0.1 (− 1.07, 4.08)	0.52
MIP-1β	0.05 (− 0.02, 0.14)	0.16	0.02 (− 0.43, 2.08)	0.80
TNF-α	0.04 (− 0.08, 0.19)	0.58	0.02 (− 2.07, 2.61)	0.88

## Discussion

Short-term exposure to ambient UFPs was robustly associated with a greater increase in the serum TxB2 level in COPD than non-COPD individuals, suggesting that COPD patients are more susceptible to UFP-associated platelet activation and pro-thrombosis. This observation could be explained, at least partially, by the greater increase in UFP-associated lipoxygenase-mediated lipid peroxidation assessed by the serum HETE levels in COPD patients.

### Susceptibility of COPD patients to UFP-associated pro-thrombosis

Our findings are consistent with Hildebrandt et al*.* [23], who reported associations between short-term exposures to ambient air PM and blood coagulation in 38 males with chronic pulmonary disease, but data on healthy controls were lacking in that study. We found that COPD and non-COPD individuals exhibited different TxB2 responses to UFPs and PM_2.5_, which provides evidence that COPD patients are more susceptible to ambient PM-associated pro-thrombosis.

Consistent with our previous studies [31, 32], UFP numbers were not correlated with PM_2.5_ concentrations. UFPs were the most abundant particles by number while they contributed little to PM_2.5_ mass concentrations. Moreover, measured UFPs has a short lifetime and is mostly originated from nearby emission and/or formation processes with greater spatial and temporal variability than PM_2.5_. In this study, the TxB2 changes with UFPs were different from those with PM_2.5_ in terms of the time course, suggesting that exposure to UFPs is associated with pro-thrombotic changes independent of PM_2.5_. Moreover, our two-pollutant models revealed that UFPs exhibited the most robust associations with the TxB2 levels, consistent with the data of previous animal and human studies indicating that smaller particulate matters are associated with greater platelet activation and thrombosis [28]. Using an in vivo hamster model, Nemmar et al*.* [17] showed that positively charged UFPs induce platelet aggregation, probably by attaching to platelet sialic acid groups and thus bridging the platelets. In another study that used the same model, intratracheal instillation of diesel exhaust particles (20–50 nm in diameter) rapidly activated circulating platelets (as assessed ex vivo using a platelet function analyser) [18]. In epidemiological studies, UFP exposure has been associated with increased levels of pro-thrombotic biomarkers in healthy individuals [33] and patients with coronary heart disease [20] and type 2 diabetes [34].

Compared to the significant TxB2 increases for 3–8 days, there was a smaller TxB2 change for 1–2 days, suggesting that UFPs may indirectly activate platelets. Consistently, a previous human study showed that particulate matter was associated with ADP-induced platelet aggregation for time lags within 72–96 h rather than 0–24 h [35]. Although it has been postulated that inhaled UFP can directly activate platelets within an hour possibly by crossing the lung epithelium into the blood circulation [36], only low levels of translocation (i.e., maximum 0.4% of the lung burden) to the kidney and liver were observed [37]. Besides, we observed that the TxB2 changes were tapering off with longer UFP exposure, which is consistent with our previous finding during the Beijing Olympics showing that PM-associated P-selectin increases were decreased with longer lags in 125 healthy young adults [38]. A fibrinolytic mechanism may be responsible for this observation. Under normal conditions, platelet activity, coagulation, and fibrinolysis are in dynamic balance. Exposure to particulate pollutants may affect each of these processes [28]. For example, a recent in vivo study showed that repeat dose exposure of particles increased tissue factor, FXa, and tissue plasminogen activator, leading to activation of both coagulation and fibrinolytic system in SD rats [39].

### Biological mechanisms underlying the susceptibility of COPD patients to UFPs

We found a possible biochemical link between UFP exposure and TxB2 increase by investigating the biomarkers of lipid peroxidation. 12-HETE and 15-HETE are generated from arachidonic acid via lipoxygenase-mediated peroxidation [40]; both were considered exquisite biomarkers of UFP-induced lipid peroxidation [41–44]. We observed significant increases in serum HETE levels associated with short-term exposure to UFPs in both COPD and non-COPD individuals. However, the associations were stronger in COPD patients, suggesting that they are more susceptible to UFP-associated pro-oxidative effects than non-COPD individuals, in agreement with our previous observations [26]. Causal mediation analyses suggested that, in COPD patients, UFP-associated increases in 12-HETE and 15-HETE levels may promote thrombosis. The 12-lipoxygenase that generates 12-HETE is an important regulator of platelet activation and thrombus formation, which can be supported by at least three postulated mechanisms [45]. An early study reported that 12-HETE activated the NADPH oxidase pathway to generate reactive oxygen species, which has been known to enhance platelet activation [46]. It was suggested that 12-HETE potentiates dense granule secretion in platelets [47]. In addition to free 12-HETE, phospholipid-esterified 12-HETE is also generated in agonist-activated human platelets and enhances tissue factor-dependent thrombin generation [48]. Besides, Vijil et al*.* found that 15-HETE increased platelet aggregation and thrombin generation in vitro [49]. Thus, the involvement of lipoxygenase-derived peroxidation explains the enhanced UFP-associated pro-thrombotic change seen in COPD patients.

Although we failed to detect any significant mediation effects of IL-8, MCP-1, MIP-1α, MIP-1β, TNF-α, and IL-1β on the association between UFP exposure and TxB2 increase, we cannot rule out the potential role of inflammatory activation in UFP-associated pro-thrombosis. In general, we found similar patterns of association between some inflammatory mediators (i.e., IL-8, MCP-1, and MIP-1α) and TxB2, supporting the hypothesis of inflammation-thrombosis crosstalk following particle exposures [28]. Consistently, Emmerechts et al. found that particle exposures over the preceding week were associated with C-reactive protein, leukocytes, and fibrinogen, as well as with tissue factor-dependent procoagulant changes in a group of patients with diabetes [50]. On the other hand, we observed a somewhat stronger increase in TNF-α, MIP-1β, and IL-1β with longer exposures when the TxB2 change was attenuated. This seems counterintuitive but could be explained by previous animal studies showing that UFP exposure caused thrombotic and inflammatory effects at different lags [18, 51, 52]. However, there is also some evidence that exposure to UFP can increase thrombus formation independent of inflammation [53], as nanoparticles may penetrate into the blood circulation with the potential for direct effects on platelet activation [54].

### Limitations

First, the present study was designed to compare the differences in TxB2 response to PM exposures between COPD patients and non-COPD controls in a real-world exposure condition. However, observational studies do not prove causality, although we observed a stronger association in the COPD patients. Second, serum TxB2 measurement is a platelet COX-1–dependent assay and it does not represent COX-1-independent platelet function that could be measured by other assays (e.g., platelet function analyzer-100 collagen-ADP closure time). However, both serum TxB2 and collagen-ADP closure time were associated with major adverse cardiovascular events [55]. Additionally, assessment of platelet TxA2 biosynthesis can be performed through measurement of urinary enzymatic metabolite 11-dehydro-TxB2 [29], which could be used for validation of our findings in further studies. Third, use of aspirin significantly impacts TxB2 levels. Our main analyses were based on all participants including users of aspirin because they accounted for a quarter of the samples in both the COPD and non-COPD groups. In the sensitivity analyses, our main results that UFP-associated TxB2 changes were significantly higher in the COPD group compared to the non-COPD group remained stable after excluding the users of aspirin (Additional file 1: Table S3). Finally, the air pollutant concentrations were measured at a monitoring station, and may thus differ from those in the homes of participants; particularly, UFP levels exhibit high spatial variation. However, assessment of personal UFP exposure is both difficult and very imprecise.

## Conclusions

This panel study showed that compared with non-COPD individuals, short-term exposure to ambient UFPs was associated with a greater increase in the serum thromboxane level among COPD patients, suggesting that UFPs may be an important risk factor for cardiovascular comorbidities in such patients. Lipoxygenase-derived peroxidation products mediated UFP-associated thromboxane change, which added new evidence that oxidative stress plays a role in mediating the pro-thrombotic effects of UFP exposures. Our findings have implications that inhibitors targeting lipoxygenase-mediated pathways may attenuate the pro-thrombotic effects of PM air pollution exposure on susceptible populations. Indeed, emerging in vivo evidence suggests that 12-lipoxygenase is a novel antiplatelet target with only a minimal risk of bleeding [45]. Further studies should investigate the underlying mechanisms by which lipoxygenase potentiates platelet activation and thrombosis following PM exposures in susceptible populations, such as those with COPD.

## Methods

### Study participants

Panel study designs that collect repeated measurements from a panel of subjects are often used to evaluate the short-term health effects of environmental exposures in environmental epidemiology. We enrolled individuals with and without COPD in a panel study on COPD in Beijing (COPDB) [56]. In 2016, COPDB recruited 135 participants aged 40–80 years from a community-based (Shi Cha Hai) population. COPD patients were diagnosed by medical professionals using the following criteria: post-bronchodilator forced expiratory volume in 1 s/forced vital capacity (FEV_1_/FVC ratio) < 0.7; respiratory symptoms; and a history of risk factors. COPD severity was classified as mild (FEV_1_ ≥ 80% predicted), moderate (50% ≤ FEV_1_ < 80% predicted), severe (30% ≤ FEV_1_ < 50% predicted), and very severe (FEV_1_ < 30% predicted), as recommended by Global Initiative for Chronic Obstructive Lung Disease (GOLD) criteria. All participants gave written informed consent at recruitment and completed baseline questionnaires exploring age, sex, the body mass index (BMI), educational level, income, smoking history, and medication use. Each participant was invited to four follow-up visits from August 2016 to September 2017. Of the 135 participants, 67 completed four visits, 28 completed three, and 17 completed two. Thirty-one participants self-reported taking aspirin before each follow-up visit and four took aspirin for at least half of the follow-up visits. The study was registered in the Chinese Clinical Trial Registry (ChiCTR1900023692) and the protocol was approved by the Institutional Review Board of Peking University Health Sciences Centre (IRB no. 00001052–15088).

### Biomarker measurements

During each visit, whole-blood samples were collected by a physician of the Shi Cha Hai Community Health Service Centre between 8:00 and 9:30 am and sera were prepared via centrifugation and stored in pro-coagulation tubes at − 80 °C. The serum concentrations of TxB2, 12-HETE, and 15-HETE were measured via online solid-phase extraction-liquid chromatography-triple quadrupole mass spectrometry (SPE-LC–MS/MS) following an established protocol [57]. Briefly, 50 µL serum was pretreated with 200 µL extraction mix containing 165 µL organic solvent, 10 µL antioxidant solution, and 25 µL deuterated internal standard mix solution. Online SPE-LC–MS/MS was performed using an Agilent 1290 Infinity Flexible Cube, an Agilent 1290 Infinity ultrahigh-performance liquid chromatography apparatus equipped with an Acquity UPLC CSH C18 column (100 × 2.1 mm, 1.7 μm; Waters, Milford, MA, USA), and an Agilent 6470 triple quadrupole (QqQ) mass spectrometer with an electrospray ionization source. Analyte standards (purities ≥ 95%) were purchased from Cayman Chemicals (Ann Arbor, MI, USA). Standard curves were generated at 12 different concentrations (from 0.0018 to 9 ng/mL). Quality control samples (pooled sera) were used to evaluate biomarker recoveries and precision. All sera were fully anonymized and randomly analyzed.

Serum concentrations of IL-1β, IL-8, MCP-1, MIP-1α, MIP-1β, and TNF-α were measured using an immunological method employing Milliplex technology (Merck & Co. Inc., Kenilworth, NJ, USA) following the manufacturer’s instructions (HCYTOMAG-60K kit) [25, 56]. Briefly, 25 µL serum and 25 µL bead suspension were mixed in the wells of 96-well microplates and incubated for 18 h at 4 °C in the dark. Antibody and streptavidin–phycoerythrin solutions were added, and subsequent analysis employed the Merck Luminex 200 platform. Detailed information on the method validation is provided in Additional file 1: Table S4.

### Air pollutant exposure

Air pollutant levels were measured at a monitoring station on the rooftop of the Shi Cha Hai Community Health Service Centre (about 12 m above ground), which lies within 3 km of the residences of at least 80% of participants [56]. Particle number concentrations were obtained at 1 min resolution using a fast mobility particle sizer spectrometer (FMPS) (Model 3091; TSI Inc., Shoreview, MN, USA). For all particle size channels, FMPS yields a normalized particle concentration (*NC* = *dN/dlogDp*) where *dN* and *dlogDp* are the concentration and the log difference in channel width. The number concentrations of UFPs were the sum of *NC* × *dlogDp* over the size range 5.6–93.1 nm. The mass concentration of particulate matter ≤ 2.5 μm in diameter (PM_2.5_) was monitored continuously at 1 h resolution using a beta-attenuation mass monitor (BAM-1020; Met One Instruments Inc., Grants Pass, OR, USA). Gaseous pollutants (nitrogen dioxide [NO_2_], sulphur dioxide [SO_2_], carbon monoxide [CO], and ozone [O_3_]) were measured hourly using online analysers (models 42i, 43c, 48i, and 49i, respectively; Thermo Scientific, Waltham, MA, USA). Temperature and relative humidity were monitored using a portable weather station attached to a four-channel aerosol sampler (TH-16A; Tianhong, Inc., Wuhan, Hubei, China).

### Statistical methods

The primary aim of the study was to assess the association between short-term exposure to air particles and serum TxB2 levels in COPD patients. Our study was designed to be adequately powered to detect a slope of 0.005 per μg/m^3^ increase in PM_2.5_ or a slope of 0.05 per 10^3^/cm^3^ increase in UFP, based on pollution levels observed in urban Beijing between 2013 and 2015 and previous studies linking air particles and TxB2 or other COX metabolites as our best estimate of what we might encounter [58, 59]. Technical details were described in the Additional file 1.

The associations between serum TxB2 concentrations and the average concentrations of air pollutants were evaluated using linear mixed-effects (LMEs) models with participant-specific random intercepts, which accounts for the correlations that arise from intraindividual repeated observations. The exposure time windows were within 14 days preceding the clinical visits, as suggested by similar studies on COPD [24, 25]. The models included age, sex, the BMI, smoking status, educational level, income, and the day of the week. Average 4-day temperatures and 14-day relative humidities preceding the clinical visits were also included. Interaction terms (COPD status and pollutant concentration) were included to explore whether the COPD and non-COPD subgroups responded differently to pollutant exposure. To investigate the UFP associations independent of co-pollutants, we used two-pollutant models that included the PM_2.5_ masses and the levels of NO_2_, SO_2_, CO, and O_3_. Sensitivity analyses were conducted with adjustment for aspirin usage, by excluding observations in cold weather, and by excluding participants with cotinine levels ≥ 50 ng/mL (from cigarettes) or TxB2 values > 67 ng/mL (> 1.5 × the interquartile range above the third quartile). To explore the potential biological mechanisms underlying UFP-associated TxB2 change, we used mediation analysis to calculate the extent by which the change in TxB2 serum concentration associated with exposure was mediated by biomarkers of lipid peroxidation and inflammation [60].

All hypothesis tests were two-sided and the significance level was set to 0.05. Undetected biomarkers were assigned with a concentration of lower limit of quantification/2 and all biomarkers were logarithmic-transformed before statistical analyses. The results are presented as percentage changes with 95% confidence intervals (CIs) of biomarker concentrations per 10^3^/cm^3^ increase in UFPs and per 10 µg/m^3^ increase in PM_2.5_ during the 1–14 days preceding the clinical visits. LME modelling and mediation analyses were performed using the “lmerTest” and “mediation” packages of R (version 3.5.3).

## Supplementary Information

**Additional file 1: Table S1**. Classification of COPD participants based on GOLD scoring. **Table S2**. Daily concentrations of ambient air particulate matter and meteorological parameters during the study period. **Table S3**. Sensitivity analyses of the association between the ultrafine particles (UFPs) level and the serum thromboxane B2 concentratio. **Table S4**. Summary of the lower limit of quantification (LLOQ), goodness of fit (R2), recovery, precision, and detection rate of each biomarker. **Table S5. **Determinants for the random-effects models and the calculated sample size.** Figure S1**. Serum thromboxane (Tx)B2 levels by COPD severity (A) and aspirin use (B).

## Data Availability

Not applicable.
