# Supplementary material for: Pro-thrombotic changes associated with exposure to ambient ultrafine particles in patients with chronic obstructive pulmonary disease: roles of lipid peroxidation and systemic inflammation
Source: Part Fibre Toxicol. 2022 Oct 24;19:65. doi: 10.1186/s12989-022-00503-9 (PMC9590143; doi:10.1186/s12989-022-00503-9)
Supplement: Supplementary file 1 — Additional file 1: Table S1. Classification of COPD participants based on GOLD scoring. Table S2. Daily concentrations of ambient air particulate matter and meteorological parameters during the study period. Table S3. Sensitivity analyses of the association between the ultrafine particles (UFPs) level and the serum thromboxane B2 concentratio. Table S4. Summary of the lower limit of quantification (LLOQ), goodness of fit (R2), recovery, precision, and detection rate of each biomarker. Table S5. Determinants for the random-effects models and the calculated sample size. Figure S1. Serum thromboxane (Tx)B2 levels by COPD severity (A) and aspirin use (B). [file 12989_2022_503_MOESM1_ESM.docx]

**ADDITIONAL FILE**

**Pro-thrombotic changes associated with exposure to ambient ultrafine particles in patients with chronic obstructive pulmonary disease: roles of lipid peroxidation and systemic inflammation**

Teng Wang^1#^, Xi Chen^1,2#^, Haonan Li^1^, Wu Chen^1^, Yifan Xu^1^, Yuan Yao^1^, Hanxiyue Zhang^1^, Yiqun Han^1,3#^, Lina Zhang^4^, Chengli Que^5^, Jicheng Gong^1^, Xinghua Qiu^1^, Tong Zhu^1*^

^1^BIC-ESAT and SKL-ESPC, College of Environmental Sciences and Engineering, Peking University, Beijing, China

^2^Hebei Technology Innovation Center of Human Settlement in Green Building (TCHS), Shenzhen Institute of Building Research Co., Ltd., Xiongan, China

^3^Environmental Research Group, MRC Centre for Environment and Health, Imperial College London, London, UK

^4^Shi Cha Hai Community Health Service Center, Beijing, China

^5^Peking University First Hospital, Peking University, Beijing, China

*Corresponding author: Tong Zhu; telephone: 010-62754789; email: tzhu@pku.edu.cn

^#^Teng Wang and Xi Chen contributed equally to this work

**Table of Contents**

Additional file 1: [Table S1. Classification of COPD participants based on GOLD scoring 3](#_Toc111471644)

Additional file 1: [Table S2. Daily concentrations of ambient air particulate matter and meteorological parameters during the study period 3](#_Toc111471645)

Additional file 1: [Table S3. Sensitivity analyses of the association between the ultrafine particles (UFPs) level and the serum thromboxane B2 concentration. 4](#_Toc111471646)

Additional file 1: [Table S4. Summary of the lower limit of quantification (LLOQ), goodness of fit (R^2^), recovery, precision, and detection rate of each biomarker. 4](#_Toc111471647)

Additional file 1: [Figure S1. Serum thromboxane (Tx)B2 levels by COPD severity (A) and aspirin use (B) 5](#_Toc111471648)

Additional [methods 6](#_Toc111471649)

# Additional file 1: Table S1. Classification of COPD participants based on GOLD scoring

| Classification of severity of airflow limitation^a^ | No. of patients with COPD |
| --- | --- |
| GOLD 1: Mild | 17 (32%) |
| GOLD 2: Moderate | 27 (51%) |
| GOLD 3: Severe | 6 (11%) |
| GOLD 4: Very Severe | 3 (6%) |

^a^ The classification uses specific spirometric cut-points recommended by Global Initiative for Chronic Obstructive Lung Disease (GOLD, 2022). Mild: FEV_1_ ≥ 80% predicted; Moderate: 50% ≤ FEV_1_ < 80% predicted; Severe: 30% ≤ FEV_1_ < 50% predicted; Very Severe: FEV_1_ < 30% predicted

# Additional file 1: Table S2. Daily concentrations of ambient air particulate matter and meteorological parameters during the study period

| Variables | N | Mean (SD) | 25th | 75th | Range |
| --- | --- | --- | --- | --- | --- |
| PM_2.5_, μg/m^3^ | 224 | 69.7 (61.1) | 31.7 | 85.8 | 10.7–475.5 |
| UFPs, 10^3^/cm^3^ | 208 | 12.5 (4.3) | 9.4 | 15.2 | 3.9–28.0 |
| Temperature, ºC | 215 | 19.4 (9.9) | 13.2 | 26.9 | -3.6–32.4 |
| Relative humidity, % | 215 | 54.2 (22.0) | 33.9 | 71.9 | 13.6–99.7 |

Abbreviations: PM_2.5_, particulate matter with aerodynamic diameter ≤ 2.5 μm; UFPs, particles with aerodynamic diameter ≤ 0.1 μm

# Additional file 1: Table S3. Sensitivity analyses of the association between the ultrafine particles (UFPs) level and the serum thromboxane B2 concentration.

| Model | COPD | | non-COPD | | *P_interaction_* |
| --- | --- | --- | --- | --- | --- |
|  | Change, %  (95% CI) | *P* | Change, %  (95% CI) | *P* |  |
| Main model | 25.4 (12.8, 39.4) | <0.001 | 11.2 (1.1, 22.3) | 0.03 | 0.01 |
| Excluding observations | | | | | |
| Aspirin use | 17.8 (8.3, 28.1) | <0.001 | 5.5 (-1.9, 13.4) | 0.15 | 0.003 |
| Cotinine ≥50 ng/mL | 17.1 (4.2, 31.6) | 0.009 | 7.7 (-2.6, 19.1) | 0.15 | 0.11 |
| TxB2 outliers | 28.2 (13.9, 44.1) | <0.001 | 10 (-0.9, 22.1) | 0.08 | 0.003 |
| Cold weather | 32.9 (14.7, 53.9) | <0.001 | 11.7 (-1.1, 26.1) | 0.08 | 0.04 |
| The 4^th^ clinical visit | 25.8 (10.9, 42.8) | <0.001 | 11.5 (-1.3, 25.8) | 0.08 | 0.02 |

# Additional file 1: Table S4. Summary of the lower limit of quantification (LLOQ), goodness of fit (R^2^), recovery, precision, and detection rate of each biomarker.

| Analyte | LLOQ  (ng/mL) | R^2^ | Recovery  (%)^a^ | Precision  (%)^b^ | Detection rate  (%)^c^ |
| --- | --- | --- | --- | --- | --- |
| TxB2 | 0.0045 | 0.998 | 87–94 | 7–8 | 97 |
| 12-HETE | 0.009 | 0.997 | 102 | 9 | 100 |
| 15-HETE | 0.0045 | 0.996 | 85-100 | 4-6 | 100 |
| IL-1β | 0.0008 | 0.999 | 97–101 | 0–11 | 66 |
| IL-8 | 0.0004 | 0.999 | 97–101 | 1–8 | 100 |
| MCP-1 | 0.0019 | 0.999 | 97–103 | 0–8 | 100 |
| MIP-1α | 0.0029 | 0.999 | 75–103 | 0–15 | 58 |
| MIP-1β | 0.0030 | 0.999 | 99–106 | 0–6 | 97 |
| TNF-α | 0.0007 | 0.999 | 99–101 | 0–8 | 100 |

^a^ The recoveries of TxB2, 12-HETE, and 15-HETE were determined in serum samples spiked at 0.1, 1, and 5 ng/mL of analyte standards, and shown as ranges across the three levels. The recoveries of IL-8, MCP-1, TNF-α, and MIP-1β were determined in standard samples spiked at seven different concentrations from 0–10 ng/mL of analyte standards, and shown as ranges across all levels.

^b^ Precision was expressed as the relative standard deviation (RSD) of the replicates for up to three levels and shown as a range across levels.

^c^ Detection rate indicates the proportion of serum samples above the LLOQ of the analyte.

LLOQ, lower limit of quantification; TxB2, thromboxane B2; HETE, hydroxyeicosatetraenoic acid; IL-8, interlukin-8; MCP-1, monocyte chemoattractant protein-1; TNF-α, tumour necrosis factor alpha; MIP-1β, macrophage inflammatory protein 1 beta

# Additional file 1: Figure S1. Serum thromboxane (Tx)B2 levels by COPD severity (A) and aspirin use (B). Very severe COPD participants were combined with the severe participants.

# Additional methods

The sample size can be estimated as (Weichenthal S et al., 2017):

$$n = \frac{{(1.96+0.84)}^{2}}{\beta^{2}}\times\frac{\sigma_{residual}^{2}}{m\times{MS}_{X}}$$

where n is the number of participants, β is the magnitude of the slope we wish to detect, σ^2^_residual_ is the within-individual variance of the response measure, m is the number of within- individual measurements, and MS_X_ is the mean squared distance between the individual’s X’s and their mean.

As shown in Table S5, the number of participants required for a random-intercept model to detect a slope of 0.005 per unit increase in PM_2.5_ (equivalent to a 5% increase in TxB2 per 10 μg/m^3^ increase in PM_2.5_), assuming four measurements per participant and an MSx value of 2500 is **45** with a type I error rate of 95% and 80% power. Similarly, we would need to have a sample size of **33** people to detect a slope of 0.05 per 1000 UFP with a type I error rate of 95% and 80% power. Notably, the residual variance was set based on previous studies linking particles and TxB2 or other COX metabolites as our best estimate of what we might encounter.

**Additional file 1: Table S5. Determinants for the random-effects models and the calculated sample size**

|  | β | σ_residual_ | m | MSx | n |
| --- | --- | --- | --- | --- | --- |
| PM_2.5_, μg/m^3^ | 0.005 | 1.2 | 4 | 2500 | 45 |
| Ultrafine particles, 10^3^ #/cm^3^ | 0.05 | 1.2 | 4 | 20 | 33 |

**Reference**

Weichenthal S, Baumgartner J, Hanley JA. Sample Size Estimation for Random-effects Models. *Epidemiology* 2017; 28(6): 817-26.
